# Is autism driven by epilepsy in infants with Tuberous Sclerosis Complex?

**DOI:** 10.1002/acn3.51128

**Published:** 2020-07-23

**Authors:** Romina Moavero, Katarzyna Kotulska, Lieven Lagae, Arianna Benvenuto, Leonardo Emberti Gialloreti, Bernhard Weschke, Kate Riney, Martha Feucht, Pavel Krsek, Rima Nabbout, Anna C. Jansen, Konrad Wojdan, Julita Borkowska, Krzysztof Sadowski, Christoph Hertzberg, Monique M. Van Schooneveld, Sharon Samueli, Alice Maulisovà, Eleonora Aronica, David J. Kwiatkowski, Floor E. Jansen, Sergiusz Jozwiak, Paolo Curatolo, the EPISTOP Consortium

**Affiliations:** ^1^ Child Neurology and Psychiatry Unit Systems Medicine Department Tor Vergata University Via Montpellier 1 Rome 00133 Italy; ^2^ Child Neurology Unit Neuroscience and Neurorehabilitation Department “Bambino Gesù” Children’s Hospital IRCCS P.zza S. Onofrio 4 Rome 00165 Italy; ^3^ Department of Neurology and Epileptology The Children's Memorial Health Institute Al. Dzieci Polskich 20 Warsaw 04‐730 Poland; ^4^ Department of Development and Regeneration‐Section Pediatric Neurology University Hospitals KU Leuven Leuven Belgium; ^5^ Department of Biomedicine and Prevention Tor Vergata University of Rome Via Montpellier 1 Rome 00133 Italy; ^6^ Department of Child Neurology Charité University Medicine Berlin Augustenburger Platz 1 Berlin 13353 Germany; ^7^ Neuroscience Unit Queensland Children’s Hospital 501 Stanley Street South Brisbane QLD 4101 Australia; ^8^ School of Clinical Medicine University of Queensland St Lucia QLD 4072 Australia; ^9^ Department of Pediatrics Medical University Vienna Vienna Austria; ^10^ Department of Paediatric Neurology Charles University Second Faculty of Medicine and Motol University Hospital Prague Czech Republic; ^11^ Department of Pediatric Neurology Reference Centre for Rare Epilepsies Necker‐ Enfants Malades Hospital University Paris Descartes Imagine Institute Paris France; ^12^ Pediatric Neurology Unit‐UZ Brussel Brussels Belgium; ^13^ Warsaw University of Technology Institute of Heat Engineering Warsaw Poland; ^14^ Transition Technologies ul. Pawia 5 Warsaw 01‐030 Poland; ^15^ Diagnose und Behandlungszentrum für Kinder und Jugendliche Vivantes Klinikum Neuköln Berlin Germany; ^16^ Department of Child Neurology Brain Center University Medical Center Utrecht Utrecht The Netherlands; ^17^ Department of (Neuro)Pathology Amsterdam UMC University of Amsterdam Amsterdam Neuroscience Meibergdreef 9 Amsterdam The Netherlands; ^18^ Stichting Epilepsie Instellingen Nederland (SEIN) Heemstede The Netherlands; ^19^ Brigham and Women's Hospital Harvard Medical School Boston MA 02115; ^20^ Department of Child Neurology Medical University of Warsaw Warsaw Poland

## Abstract

**Objective:**

To evaluate the relationship between age at seizure onset and neurodevelopmental outcome at age 24 months in infants with TSC, as well as the effect on neurodevelopmental outcome of early versus conventional treatment of epileptic seizures with vigabatrin (80–150 mg/kg/day).

**Methods:**

Infants with TSC, aged ≤4 months and without previous seizures were enrolled in a prospective study and closely followed with monthly video EEG and serial standardized neurodevelopmental testing (Bayley Scales of Infant Development and Autism Diagnostic Observation Schedule).

**Results:**

Eighty infants were enrolled. At the age of 24 months testing identified risk of Autism Spectrum Disorder (ASD) in 24/80 children (30.0%), and developmental delay (DD) in 26/80 (32.5%). Children with epilepsy (51/80; 63.8%) had a higher risk of ASD (*P* = 0.02) and DD (*P* = 0.001). Overall, no child presented with moderate or severe DD at 24 months (developmental quotient < 55). In 20% of children abnormal developmental trajectories were detected before the onset of seizures. Furthermore, 21% of all children with risk of ASD at 24 months had not developed seizures at that timepoint. There was no significant difference between early and conventional treatment with respect to rate of risk of ASD (*P* = 0.8) or DD (*P* = 0.9) at 24 months.

**Interpretation:**

This study confirms a relationship between epilepsy and risk of ASD/DD. However, in this combined randomized/open label study, early treatment with vigabatrin did not alter the risk of ASD or DD at age 2 years.

## Introduction

Tuberous Sclerosis Complex (TSC) is a multisystem disorder caused by a mutation in the *TSC1* or *TSC2* gene, associated with intellectual disability or autism spectrum disorder (ASD) in 40–50% of affected individuals.^1^, ^2^ Failure of *TSC1* or *TSC2* gene products hamartin/tuberin to suppress mTORC1 may lead to alterations in brain development, as well as abnormal cell growth and differentiation interfering with synaptogenesis and balance of excitation/inhibition.^3^ Dysregulated mTOR signaling has also been associated with aberrant connectivity supporting the assumption of intrinsic epileptogenicity in individuals with TSC and a possible association with cognitive impairment and ASD.^4^

Due to the presence of epileptogenic cortical tubers, up to 85% of individuals with TSC develop epilepsy, with seizure onset during the first year of life in 67% of cases.^5^, ^6^ Epilepsy has previously been considered to be a causal factor for subsequent TSC associated neuropsychiatric disorders (TAND) including ASD and intellectual disability (ID),^7^ but it is actually considered more as a risk factor for a worse long‐term outcome.^8^ TSC‐associated ASD is a multifactorial condition, influenced by many risk factors including early seizure onset, infantile spasms, persistent and frequent seizures, longer treatment lag to antiepileptic drug treatment, and temporal interictal epileptiform discharges (IED) on the electroencephalogram (EEG).^9^, ^10^, ^11^

Prenatal or early postnatal diagnosis of TSC, before the onset of seizures, is currently possible in a growing number of cases, by detecting cardiac rhabdomyomas and cortical tubers.^12^ Therefore, close follow‐up of all newborns/infants with TSC using repeated standardized neurodevelopmental tests and video EEG recordings at key developmental time points has been strongly recommended.^13^, ^14^, ^15^, ^16^ It has been shown that IED may correctly predict the onset of clinical seizures^17^, ^18^ and that early detection and prompt control of seizures can mitigate the severity of long‐term neurodevelopmental outcomes, with lower rates of ID and ASD.^6^, ^19^ In a small prospective single center nonrandomized clinical trial, antiepileptic treatment with Vigabatrin, given early when IED were first detected on EEG, improved long‐term epilepsy and developmental outcomes compared to when Vigabatrin treatment was started after the onset of clinical seizures.^20^, ^21^ However, to what degree seizures play a causative role for later ASD/DD, and if early treatment has the potential to prevent the subsequent development of ASD/DD remains unclear.

The aim of this study was to examine the temporal relationship between the onset of seizures and the early detection of abnormal developmental trajectories, suggestive of risk of subsequent ASD/DD in infants with TSC. A further objective was to investigate, in a longitudinal controlled trial, whether Vigabatrin treatment initiated early, before the first seizures and immediately after the first appearance of IED on EEG (early treatment), could improve neurodevelopmental outcome at 24 months compared to Vigabatrin initiation after first seizure (conventional treatment).

## Materials and Methods

### Participants and EPISTOP study design

From 1 November 2013 to 31 August 2016, male or female infants with a definite diagnosis of TSC,^22^ aged < 4 months, with no seizures reported by caregivers and with no previous AED treatment, were enrolled in the Long‐term, prospective study evaluating clinical and molecular biomarkers of EPIleptogenesiS in a genetic model of epilepsy – Tuberous SclerOsis ComPlex (EPISTOP ‐ NCT02098759, clinicaltrials.gov) study, a prospective study aimed at evaluating efficacy of Vigabatrin treatment initiated after the appearance of seizures (conventional group) or after first recorded IED on EEG before the appearance of seizures (early/preventive group). Exclusion criteria were lack of definite TSC diagnosis, prior epileptic seizure, prior antiepileptic treatment, and any condition considered by the investigator to hinder participation in the study.

Initially, a randomized controlled trial (RCT) was planned at all clinical sites; however due to Ethics Committee approval at some sites, participants at those centers were enrolled instead in a parallel open‐label trial (OLT), with treatment determined according to local clinical practice. Subjects at two sites received preventive treatment and at two others conventional treatment. In both the RCT and OLT the criteria for preventive and/or conventional treatment, IED diagnosis as well as EEG scoring system were identical. At all sites subjects with clinical and/or electrographic seizures received vigabatrin treatment without being randomized.

### Randomization and masking

In the RCT, computerized randomization was performed centrally using variably sized permuted blocks stratified for study sites. The local EEG reader sent EEG scores to the central reader, and the central reader (LL) randomly allocated eligible patients to either preventive or conventional treatment. The treating physicians and subjects’ caregivers were blinded to EEG data, and did not know whether the treatment was randomly allocated after IED or started conventionally because of seizures captured on video EEG.

Neuropsychologists performing neuropsychological tests and electrophysiologists assessing EEGs were blinded to treatment allocation and clinical history of the patients.

### Neurodevelopmental assessment

All children underwent serial neurodevelopmental assessment starting at age 6 months, and repeated at key developmental timepoints (12, 18, and 24 months) with the Bayley Scales of Infant Development third edition (BSID‐III). BSID contains items specifically designed to identify young children at risk for developmental delay, and it has been used to measure the developmental level in cognitive, motor and language domains. Children with a BSID cognitive quotient value below 70 at 24 months were classified as developmental delay (DD).

The Autistic Diagnostic Observation Schedule (ADOS) is considered the gold standard for assessing and diagnosing autism. ADOS was performed at 12 months and every six months thereafter up to the age of 24 months. At 24 months, children were classified as at risk of ASD or not based on the ADOS score (Toddler Module). At 24 months, if the ADOS was not able to be performed due to the child being nonverbal, having cognitive development below a 12‐month level or not walking independently, DSM‐5 criteria for ASD were applied. Non‐verbal children with a total score of 0–9, 10–13, and over 13 were classified as “no risk of ASD,” “mild/moderate risk of ASD,” and “high risk of ASD,” respectively. For verbal children the cut‐off scores for the three risk categories were 0–7, 8–11, and over 11.

Tests were performed by neuropsychologists certified through formal reliability training at each site and sent anonymized (identified by subject code only) to a central research team for analysis. Each center used the validated and translated version of BSID and ADOS for their own country.

At the beginning of the study all neuropsychologists had face‐to‐face training to explore and compare the different evaluating methods. Furthermore, the first tests were scored twice, first by the local neuropsychologists and then by a centralized review through a video. Possible score differences were discussed and reassessed. However, this procedure was not assessed in terms of formal inter‐rater reliability.

### Neurophysiological assessment and epilepsy management

During the first 6 months of life video EEGs were recorded for about 1 h every 4 weeks, and then every 6 weeks up to the age of 12 months, and every 8 weeks thereafter. At each visit, data were gathered on the occurrence and frequency of clinical seizures captured by the subjects’ caregivers in seizure diaries, as well as the effect and safety of (eventual) antiepileptic treatment. If focal IED were observed in more than 10% of the recording time, or multifocal or generalized IED were present 17, infants were assigned either to early treatment with Vigabatrin (80–150 mg/kg/day) or treatment was postponed until the appearance of a first seizure. Epilepsy was considered as refractory when seizures failed control with two AEDs at any time during the study.^23^

### Statistical analysis

Comparisons between groups were examined, as appropriate, by means of Welch two‐sample *t*‐test, as well as by standard nonparametric statistics, such as Pearson’s chi‐squared test and Fisher’s Exact test, Kruskal–Wallis rank sum test, equality of medians test, Spearmans’s rho and Wilcoxon rank sum test with continuity correction.

We used the Kaplan–Meier procedure to estimate time‐to‐event models in order to examine seizures and EEG abnormalities not only in terms of presence/absence (cross‐sectional) across the different groups, but also in terms of distribution of times between groups. The first seizure was used as a status variable, relating it to the time at which the seizure appeared. Risk of ASD (Yes/No), DD (Yes/No), and mutational status (TSC1/TSC2) were consecutively entered as factor variables. Log Rank (Mantel‐Cox) statistics was used to test the equality of the survival distributions for the different levels of the given factor.

Uni‐ and multivariate logistic regression models with ASD/no ASD or DD/no DD as dichotomous outcome variables were used to estimate the odds ratios (OR) of several possible explanatory covariates (type of treatment, history of epilepsy, sex, type of mutation, presence of refractory epilepsy). Models were estimated using block entry of variables, which were chosen starting from those variables that presented the lowest *P*‐values according to the univariate analyses. Independent variables were either interval level or ordinal. Logistic regression coefficients were used to estimate odds ratios for each of the independent variables in the model. To evaluate the model we used the Hosmer–Lemeshow goodness‐of‐fit statistic, which in this context appeared to be more robust than the traditional goodness‐of‐fit statistic used in logistic regressions as several of the models included continuous covariates and the overall sample size was relatively small.

Statistical significance was set at an alpha level = 0.05 and *P*‐values were reported without adjustment for multiple comparisons; adjustments for multiple comparison were made using the False Discovery Rate and no changes in results were observed. All analyses were conducted using SPSS® statistical software (IBM SPSS Statistics for Windows, Version 25.0. Armonk, NY: IBM Corp.).

## Results

### Patients’ demographics and clinical characteristics

**Figure 1:**
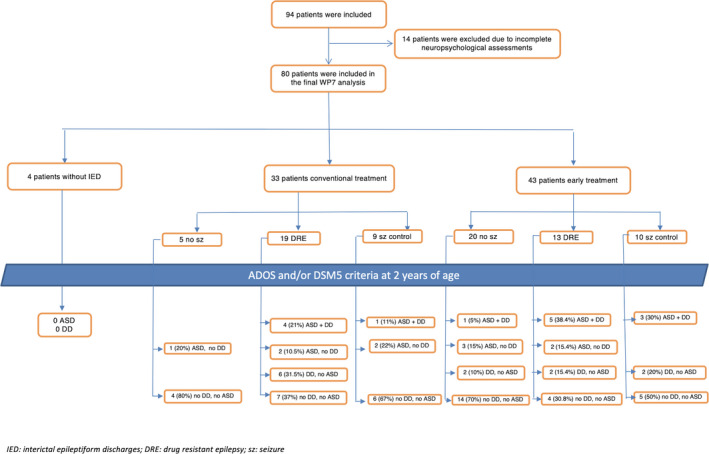
Flow‐chart showing patients’ disposition. ASD indicates risk of ASD.

Overall, 94 infants were enrolled into EPISTOP. In 14 neurodevelopmental assessments were incomplete and were therefore excluded from our substudy of EPISTOP (Fig. 1). Of the 80 children (45 M, 35 F) available for final analysis, 20 (25.0%) had mutations in *TSC1* and 59 (73.7%) in *TSC2*. In 1 child no mutation was identified. Mean age at enrollment was 34 days (range 0–120 days; median 25.5 days). At 24 months of age 51/80 (63.8%) had developed epilepsy, which was refractory in 32/51 (62.7%). Infantile spasms had occurred in 10/80 infants (12.5%). Among the 51 children with epilepsy, the mean age at seizure onset was 35 weeks (range 2–103 weeks; median 28 weeks).

The mean cognitive DQ of the 80 enrolled infants at the age of 2 years was 77.4, with 54/80 children (67.5%) showing normal cognitive development. Risk of ASD at 24 months was identified in 24/80 (30.0%) children; 14/24 also had DD, while 10/24 had a normal cognitive developmental quotient. The clinical characteristics of the whole cohort of 80 infants are summarized in Table 1.

**Table 1:** Main clinical characteristics of the 80 infants prospectively evaluated in this study.

		Overall	Early treatment (*n* = 43)	Conventional treatment (*n* = 33)
Genetics	*TSC1* mutation	20/80 (25%)	8/43 (18.6%)	10/33 (30.3%)
	*TSC2* mutation	59/80 (73.75%)	34/43 (79.1%)	23/33 (69.7%)
	No mutation identified	1/80 (1.25%)	1/43 (2.3%)	0
Epilepsy	History of epilepsy	51/80 (63.8%)	23/43 (53.5%)	28/33 (84.8%)
	Mean age at seizure onset	25 weeks	27	24
	Infantile spasms	10/51 (19.6%)	0	10/33 (30%)
	Refractory epilepsy	32/51 (62.7%)	13/43 (30.2%)	19/33 (57.6%)
	No history of epilepsy	29/80 (36.2%)	20/43 (46.5%)	5/33 (15.1%)
Neurodevelopmental status	Mean cognitive DQ	77.4	74.1	77.6
	Normal cognitive DQ	54/80 (67.5%)	28/43 (65.1%)	22/33 (66.7%)
	Mean cogDQ	86.9	83.2	87
	Cognitive DQ < 70	26/80 (32.5%)	15/43 (34.9%)	11/33 (33.3%)
	Mean cogDQ	58	57.7	58.6
	Cognitive DQ < 55	0	0	0
	Risk of ASD identified	24/80 (30%)	14/43 (32.6%)	10/33 (30.3%)
	Mean ADOS score	7.8	8.6	7.9

### Longitudinal evaluation at developmental key time points (6, 12, 18, 24 months)

**Figure 2:**
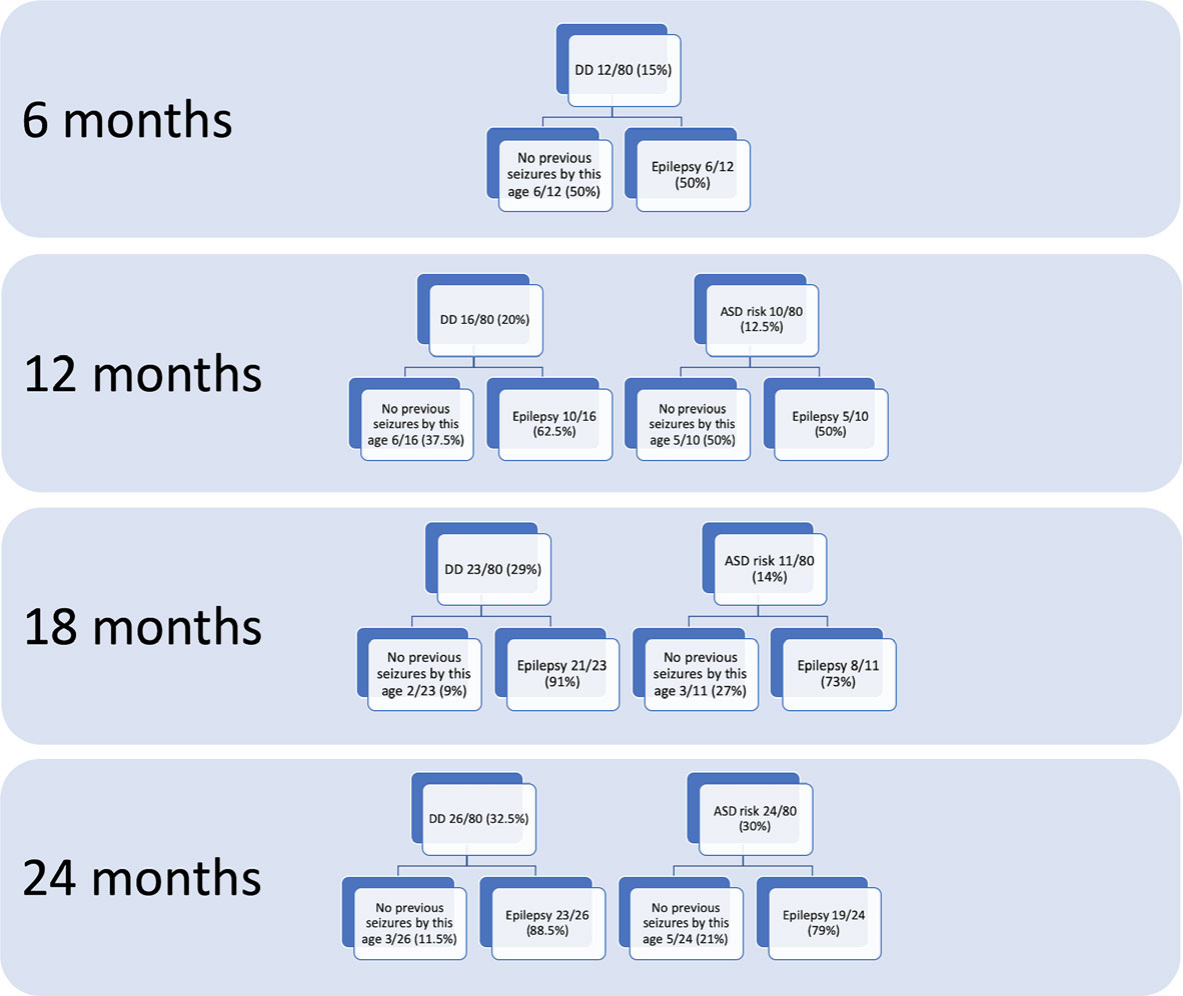
Longitudinal data show risk of ASD and cognitive developmental quotient in relation to history of epilepsy at each timepoint in the 80 children enrolled in this study.

Neurodevelopmental and epilepsy data of the 80 infants are summarized in Figure 2. The first neurodevelopmental evaluation performed at 6 months of age revealed DD in 12/80 (15.0%) children, 6/12 (50.0%) had no history of seizures to that age. At age 12 months, risk of ASD was identified in 10 children, 5/10 (50.0%) had no history of seizures to that age. DD was present in 16/80 children (20.0%); 6/16 (37.5%) had no history of seizures to that age. At age 18 months, risk of ASD was identified in 11 (13.8%) children, 3/11 (27.3%) had no history of seizures to that age. DD was present in 23/80 (28.8%) children, 2/23 (8.7%) had no history of seizures to that age.

Finally, at age 24 months, risk of ASD was identified in 24 (30%) children, 5/24 (21.0%) had no history of seizures to that age. DD was present in 26/80 (32.5%) children, 3/26 (11.5%) had no history of seizures to that age.

At 24 months of age a total of 24/80 (30.0%) children presented with risk of ASD, 14/24 (58.3%) also had DD. The ADOS could not be performed in 2 children at this age. The main clinical characteristics of the 24 children (16 M, 8 F) with risk of ASD at 24 months of age are summarized in Table 2. Eighteen (75.0%) had a *TSC2* mutation and 6 (25.0%) had a *TSC1* mutation. Nineteen (79.2%) had a history of previous seizures, 2/19 had a history of infantile spasms. Mean age at seizure onset was 30 weeks (range: 2–87 weeks; median 17 weeks). Five (21%) had no previous seizures to that age, all these children had received early Vigabatrin treatment.

**Table 2:** Main clinical characteristics of the 24 children identified with risk of ASD at the age of 24 months.

	TSC gene mutation	Age at seizure onset (weeks)	Infantile spasms	Refractory epilepsy	Delay in cognitive development	ADOS score/ASD risk	Treatment
1	TSC1	No seizures	No	No	Y	10/mild‐moderate	Early
2	TSC1	No seizures	No	No	No	8/mild‐moderate	Early
3	TSC2	No seizures	No	No	No	19/high	Early
4	TSC1	No seizures	No	No	No	21/high	Early
5	TSC2	No seizures	No	No	No	17/high	Early
6	TSC2	9	No	Yes	Y	26/high	Early
7	TSC2	87	No	No	Y	20/high	Early
8	TSC2	86	No	No	Y	15/high	Early
9	TSC2	8	No	Yes	Y	18/high	Early
10	TSC2	53	No	Yes	No	9/mild‐moderate	Early
11	TSC2	52	No	No	Y	16/high	Early
12	TSC2	51	No	Yes	Y	22/high	Early
13	TSC2	38	No	Yes	Y	high	Early
14	TSC1	2	No	Yes	No	11/mild‐moderate	Early
15	TSC2	13	No	Yes	Y	High	Early
16	TSC2	9	Yes	Yes	Y	20/high	Conventional
17	TSC2	9	No	Yes	Y	13/mild‐moderate	Conventional
18	TSC1	35	No	No	No	17/high	Conventional
19	TSC2	34	Yes	No	Y	17/high	Conventional
20	TSC2	32	No	No	No	10/mild‐moderate	Conventional
21	TSC2	17	No	Yes	Y	10/mild‐moderate	Conventional
22	TSC2	15	No	Yes	Y	15/high	Conventional
23	TSC1	14	No	Yes	No	21/high	Conventional
24	TSC2	10	No	Yes	No	12/mild‐moderate	Conventional

Of the 14/24 children with both risk of ASD and DD at 24 months of age, 7/14 (50.0%) had a decrease in cognitive DQ at this age, which had been normal earlier. All of these 7 children had a *TSC2* mutation; their mean age at seizure onset was 21 weeks (range 8–52), and all but one had refractory epilepsy. Four of them had been treated early, 3 conventionally.

Overall, 12/80 (15.0%) children were diagnosed with DD with no risk of ASD identified at 24 months of age. Of these infants, 2/12 had no epilepsy (both received early Vigabatrin treatment) while 10/12 had epilepsy (four received early treatment, six conventional treatment). The epilepsy was refractory in 8/10 (two received early treatment, six conventional treatment). All children had a cognitive developmental quotient higher than 55 at the age of 24 months. A univariate logistic regression estimated that a diagnosis of DD (independent variable) significantly increased the chance of having risk of ASD (dependent variable) at age 24 months (OR = 4.77; 95%CI: 1.73–13.16). Using a multivariate logistic model with the addition of a second independent variable, that is, type of treatment (early/preventive or conventional), the result did not change significantly (OR = 4.35; 95%CI: 1.57–12.05).

### Relationship of neurodevelopmental outcome and epilepsy at 24 months

#### History of seizures

In this cohort of infants, scores of at risk of ASD were more frequent in children with a history of epilepsy (19/51; 37.3%) than in those without a history of epilepsy (5/29; 17.2%). However, this difference failed to reach statistical significance (Chi‐Square = 2.92; *P* = 0.06). A logistic regression analysis confirmed that a history of epilepsy did not significantly increase the likelihood the child scored at risk of ASD at age 24 months (OR = 2.47; 95%CI: 0.86–7.11). Nevertheless, children with epilepsy presented with more severe ASD symptoms, confirmed by significantly higher scores on ADOS when compared to those without epilepsy (9.0 vs. 5.4; *t* = 2.30; *P* = 0.02; Mann–Whitney *U* = 710.0; *P* = 0.038).

DD was present in 23/51 (45.1%) children with a history of epilepsy versus 3/29 (10.3%) of those without epilepsy (Chi‐Square = 11.21; *P* = 0.001). Children with a history of epilepsy also had significantly lower values of cognitive DQ at 24 months when compared to children without epilepsy (72.5 vs. 86.4; *t* = 3.82; *P* < 0.001). This was also confirmed by a univariate logistic regression analysis, which estimated that a history of epilepsy (independent variable) increased the risk of the dependent variable DD (OR = 7.67; 95%CI: 2.06–28.48). After adding in a multivariate logistic model two independent variables, sex and type of mutation, epilepsy continued to be a significant risk factor for DD (OR = 6.56; 95%CI: 1.51–22.45). In another multivariate logistic regression model, which included a history of epilepsy and type of treatment (conventional or early) as independent variables, the risk of DD (dependent variable) was slightly higher, even though the confidence interval was still large (OR = 8.22; 95%CI: 2.06–32.78).

#### History of infantile spasms

The majority (41/51; 80.4%) of children who developed epilepsy in the first 2 years of life had focal seizures alone, while 10/51 (19.6%) had infantile spasms with focal seizures. Scores consistent with risk of ASD were found in 2/10 (20.0%) children with infantile spasms versus 22/41 (53.7%) of those with focal seizures alone (Chi‐Square = 3.84; *P* = 0.05). DD was detected in 5/10 (50.0%) children with infantile spasms versus 18/41 (43.9%) of those with focal seizures only (Chi‐Square = 1.76; *P* = 0.72).

#### Age at seizure onset

The mean age at seizure onset was 38 weeks in children who did not score at risk of ASD at 24 months of age, versus 30 weeks in those with risk of ASD (*t* = 1.06; *P* = 0.297). The test of equality of survival distributions for the different levels of ASD was not statistically significant (Chi‐Square = 0.76; *P* = 0.38). Children with DD at 24 months of age had earlier seizure onset (mean 30 weeks) when compared to those with normal cognitive DQ (mean 39 weeks), but this difference was not statistically significant (*t* = 1.29; *P* = 0.203), and the test of equality of survival distributions for the different levels of DD was not statistically significant (Chi‐Square = 1.16; *P* = 0.28).

To assess a possible relationship between age at epilepsy onset and neurodevelopmental status at 24 months of age, we used two different cut‐offs for age at seizure onset: 3 months (12 weeks) and 12 months (52 weeks). In this cohort of infants, no statistically significant associations were found (Table 3). Nonetheless, we observed a moderate negative correlation between age of first seizure and ADOS score (Spearman’s *r* = −0.364, *P* = 0.03): the earlier the seizure onset, the higher the ADOS score. On the other side, there was no statistically significant correlation between age at first seizure and cognitive DQ (Spearman’s *r* = 0.245; *P* = 0.083). In this study, 22 infants had epilepsy onset during the first 6 months of life, before first neurodevelopmental evaluation. Fourteen infants had epilepsy onset between 6 and 12 months of age of whom 2/14 had DD identified prior to epilepsy onset on first neurodevelopment evaluation at 6 months of age. Epilepsy onset was between 12 and 18 months in 9 infants, 2/9 had risk of ASD detected prior to epilepsy onset and 3/9 had DD detected prior to epilepsy onset on evaluations at 12 months of age. Finally, 6 infants had epilepsy onset between 18 and 24 months of age; 1/6 was identified as having risk of ASD and 1/6 to have DD on evaluations done prior to onset of the epilepsy.

**Table 3:** Prevalence of ASD and DD in 51 children with epilepsy, grouped according to the age at seizure onset.

	Epilepsy	No epilepsy	*P*‐value
ASD	19/51 (37.3%)	5/29 (17.2%)	0.06
DD	23/51 (45.1%)	3/29 (10.3%)	0.001
	Seizure onset 0–12 weeks	Seizure onset >12 weeks	*P*‐value
ASD	6/11 (54.5%)	13/40 (32.5%)	0.18
DD	7/11 (63.6%)	16/40 (40.0%)	0.16
	Seizure onset 0–12 months	Seizure onset >12 months	*P*‐value
ASD	16/38 (42.1%)	3/13 (23.1%)	0.22
DD	20/38 (52.6%)	3/13 (23.1%)	0.06
	Drug responsive epilepsy	Refractory epilepsy	*P*‐value
ASD	6/19 (31.6%)	13/32 (40.6%)	0.5
DD	6/19 (31.6%)	17/32 (53.1%)	0.13

#### Response to antiepileptic treatment

Nineteen out of the 51 children with epilepsy (37.3%) responded to antiepileptic treatment, while 32 (62.7%) had refractory epilepsy. Response to anti‐epileptic treatment did not significantly decrease risk of ASD at 24 months. Risk of ASD was detected in 13/32 (40.6%) children with refractory epilepsy versus 6/19 (31.6%) of those with controlled epilepsy (Chi‐Square = 0.46; *P* = 0.5). A logistic regression analysis failed to demonstrate a statistically significant increased risk for ASD in children with refractory epilepsy (OR = 2.17; 95%CI 0.83–5.65).

DD was diagnosed in 53.1% (17/32) of children with refractory epilepsy versus 31.6% (6/19) of those with controlled epilepsy (Chi‐Square = 2.84; *P* = 0.13). At a univariate logistic regression analysis, children with refractory epilepsy (independent variable) had a 5‐fold higher risk of DD (OR = 5.16; 95%CI: 1.90–14.05). After adjusting, in a multivariate logistic model, for sex and type of mutation, refractory epilepsy continued to be a significant risk factor for DD (OR = 4.27; 95%CI: 1.53–11.87.

### Early versus conventional Vigabatrin treatment

Only four of the 80 children (5.0%) never developed EEG abnormalities during the first 24 months of life. The remaining 76 patients therefore received early (43 infants) or conventional treatment (33 infants). Seizure onset was later in children receiving early treatment (27 weeks ± 30) compared to those receiving conventional treatment (24 weeks ± 19) (*P* = 0.08). At the age of 24 months, 10/33 children (30%) in the conventional treatment group had experienced infantile spasms, compared with none in the early group (Chi‐Square = 34.22; *P* < 0.001). Nineteen patients in the conventional group (57.6%) and 13/43 (30.2%) in the early group had refractory epilepsy (Chi‐Square = 5.74; *P* = 0.02). In this cohort of infants, all of whom had close clinical and EEG surveillance up to 24 months of age, risk of ASD was identified in 14/43 (32.6%) children treated early, and in 10/33 (30.3%) of children treated conventionally (Chi‐Square = 0.01; *P* = 0.8). The mean cognitive DQ was 77.6 (range: 55–105) and 74.1 (range: 55–105) in the conventional and early groups, respectively. DD was present in 15/43 (34.9%) children who received early treatment and in 11/33 (33.3%) children who received conventional treatment (Chi‐Square = 0.10; *P* = 0.9).

## Discussion

In this cohort of infants with TSC, prospectively evaluated during early development and closely followed by clinical and EEG examinations, children with a history of epilepsy were found to have a higher risk of ASD and lower DQ at 24 months of age when compared to children without epilepsy, consistent with other studies.^24^, ^25^ Furthermore, DD at 24 months of age was significantly related to history of epilepsy, especially refractory epilepsy.

Although our data showed a five times increased risk of DD for patients with refractory epilepsy, we were not able to show significant associations between age of epilepsy onset, presence of refractory epilepsy or early versus conventional treatment and presence of DD/ASD at 24 months of age. In our study, an earlier age at seizure onset did moderately correlate with higher total ADOS scores at 24 months of age; such purely quantitative association may suggest that earlier seizures could play a role in increasing ASD severity, even if not in determining ASD risk.

Although we found an association between a history of previous seizures and DD and severity of ASD symptoms at 24 months, 21% of the infants found to have risk of ASD and 11% of the infants with DD at 24 months had no history of previous seizures. Furthermore, some children were identified as having risk of ASD or DD before seizure onset occurred.

In our cohort, history of refractory epilepsy did not increase the risk of ASD at 2 years of age, consistent with some previous preclinical and clinical data. Several mouse models have shown neurodevelopmental deficits in the absence of seizures^26^, ^27^; ASD and DD in the absence of epilepsy have also been rarely described in retrospective human studies of young children with TSC.^5^, ^10^, ^28^ In a recent prospective study, Farach et al observed that infants with a *TSC2* pathogenic variant had significantly lower developmental scores at age 24 months, independent of seizures.^29^ In this study, early Vigabatrin treatment initiated before seizure onset did not significantly decrease the risk of ASD at 24 months, when compared to conventional treatment.

Capal and coworkers, performing the Autism Observation Scale for Infants (AOSI) at 12 months and ADOS‐2 at 24 and 36 months, noted that infants without seizures in the first year of life performed better compared to children with seizures, suggesting that an early seizure onset in the first 12 months of life can be a predictor for subsequent ASD.^24^ Nonetheless, they also observed that seizure frequency during the first 6 months of life evaluated through parents’ diary did not correlate with lower developmental scores at 24 months, and that better seizure control and reduced seizure frequency at later time points compared to earlier time points did not significantly correlate with improved developmental testing scores.^24^

Early identification of an abnormal trajectory in social communication skills and a global developmental impairment is usually present by the age of 9 months.^30^, ^31^, ^32^ However, TAND in infancy and childhood are often underdiagnosed and undertreated,^2^ and most previous studies aiming to investigate the relationship between epilepsy and early development in TSC lack accurate and objective phenotypic characterization of neurodevelopment in infancy at seizure onset.

In our cohort, we identified a clear decline of DQ in some children identified with risk of ASD at 2 years of age. More than the history of seizures per se, refractory epilepsy has been reported to increase the likelihood of a delay or arrest in cognitive development and thus it is possible that early and persistent seizure activity could additionally contribute to long‐term cognitive outcome.^25^ Abnormalities in early white matter development have been found in patients with TSC who developed ASD.^33^ Refractory epilepsy with persistent seizures may aggravate the neurodevelopmental phenotype, disrupting white matter connectivity, especially in brain circuits involved in social behavior and cognition.^34^ Therefore, seizures may represent a superimposed injury to an already abnormal myelogenic process.^35^

In the Tuberous Sclerosis 2000 Study, a prospective longitudinal study in 125 children (age 0–16 years), the authors found that severe early onset epilepsy appeared to be strictly associated with intellectual disability.^36^ Age at seizure onset was identified as the only independent risk factor for cognitive impairment in TSC.^37^ In our cohort, with close clinical and EEG surveillance of all children potentially allowing earlier and better response to seizures for all patients, age at seizure onset was not significantly associated with DD nor DQ at 24 months.

In our cohort no child had moderate or severe DD at 2 years of age, whereas this was found in around 30% of patients in a recent long term follow up study.^25^ A longitudinal study that aimed to investigate cognitive development in relation to the onset and duration of infantile spasms showed clinical evidence of impaired intelligence following infantile spasms.^38^ A more severe cognitive phenotype, with a rate of intellectual impairment of up to 70%, in infants with early onset epilepsy was related to late detection of infantile spasms and a long treatment lag.^39^ A reduced rate of severe and profound cognitive impairment, as well as a lower rate of infantile spasms than expected, in this study may therefore reflect the close surveillance of infants in this cohort, and subsequent earlier identification and treatment of spasms with Vigabatrin.^40^

Our results suggest that developmental and epileptic co‐morbidities of TSC are distinct, with both being a consequence of the genetic mutation and overactivity of the mTOR pathway present from fetal life.^41^ Mutations in TSC genes may have direct consequences for synaptic dysfunction and may directly contribute to both epilepsy and ASD/DD pathogenesis.^42^ Furthermore, altered neural connectivity may be evident as early as 12 months of age in infants with TSC who later develop ASD.^43^ Neuronal mTOR hyperactivity influences the severity of epilepsy and associated neuropathology in experimental models of TSC.^44^ Uncontrolled and recurrent seizures could contribute a superimposed and additional hit, further altering neuronal activity and connectivity over an existing abnormality due to TSC gene mutation and mTOR hyperactivity. Further prospective and longitudinal studies of larger populations of TSC infants will be required to clarify the degree to which these developmental and epileptic components, jointly or independently, contribute to shaping the final neurodevelopmental outcome of infants with TSC.

Our study has some limitations. The diagnostic power of neurodevelopmental assessment at 24 months of age is not absolute, particularly in terms of stability of diagnostic classification. It is possible that some children with no clear risk of ASD or developmental disability at 24 months of age could develop these later in life. A longer follow‐up with neurodevelopmental assessment to the age of 6 years is planned for the whole EPISTOP cohort. Furthermore, our cohort was relatively small and, consequently, it was difficult to analyze the individual impact of epilepsy onset and duration, mutational status, and duration of treatment lag on developmental outcome. Only a long‐term assessment in a large cohort of infants could disentangle different influencing factors.

In addition, the severity of ASD symptoms evaluated at 2 years of age could be influenced by early intervention therapies, which were not identical across different centers. No measure of intervention programs was included in assessing developmental outcomes in our study. Finally, it is important to note that all patients in this study had high frequency serial EEG and clinical reviews, thus allowing prompt response to any seizure/development concerns, therefore these data would not necessarily apply to infants who are not under the same close surveillance and could explain the differences observed between this study and historical cohorts.

In conclusion, we found an association between history of epilepsy and ASD/DD severity at 24 months of age. However, early serial ASD screening and neurodevelopmental evaluation identified a deviation in developmental trajectory before onset of seizures, suggesting that, at least in some individuals, epilepsy was not the sole contributor to developmental outcome.

## Authors’ Contribution

Romina Moavero, Katarzyna Kotulska, Sergiusz Jozwiak, and Paolo Curatolo contributed to conceptualization. Romina Moavero, Arianna Benvenuto, Leonardo Emberti Gialloreti, Bernhard Weschke, Kate Riney, Floor Jansen, Martha Feucht, Pavel Krsek, Rima Nabbout, Anna Jansen, Konrad Wojdan, Julita Borkowska, Krzystof Sadowski, Christoph Hertzberg, Monique M. Van Schooneveld, Sharon Samueli, Alice Maulisovà, Eleonora Aronica, David Kwiatkowski, Lieven Lagae, Sergiusz Jozwiak, and Paolo Curatolo contributed to data curation. Leonardo Emberti Gialloreti contributed to formal analysis. Katarzyna Kotulska and Sergiusz Jozwiak contributed to funding acquisition. Romina Moavero contributed to investigation. Leonardo Emberti Gialloreti contributed to methodology. Sergiusz Jozwiak contributed to project administration. Leonardo Emberti Gialloreti and Paolo Curatolo contributed to supervision. Romina Moavero and Paolo Curatolo contributed to writing – original draft. Romina Moavero, Arianna Benvenuto, Leonardo Emberti Gialloreti, Bernhard Weschke, Kate Riney, Floor Jansen, Martha Feucht, Pavel Krsek, Rima Nabbout, Anna Jansen, Konrad Wojdan, Julita Borkowska, Krzystof Sadowsk, Christoph Hertzberg, Monique M. Van Schooneveld, Sharon Samueli, Alice Maulisovà, Eleonora Aronica, David Kwiatkowski, Lieven Lagae, Sergiusz Jozwiak, and Paolo Curatolo contributed to writing – review & editing.

## Conflicts of Interest

The authors declare no conflict of interest.
